# Effect of glycine pretreatment on the shear bond strength of a CAD/CAM resin nano ceramic material to dentin

**DOI:** 10.4317/jced.52630

**Published:** 2016-04-01

**Authors:** Matteo Ceci, Marco Pigozzo, Andrea Scribante, Riccardo Beltrami, Marco Colombo, Marco Chiesa, Claudio Poggio

**Affiliations:** 1 Department of Clinical-Surgical, Diagnostic and Pediatric Sciences, Section of Dentistry, University of Pavia, Pavia, Italy; 2 Department of Brain and Behavioral Sciences, Section of Statistics, University of Pavia, Pavia, Italy

## Abstract

**Background:**

The purpose of this study was to evaluate the effect of glycine pretreatment on the shear bond strength between dentin and a CAD/CAM resin nano ceramic material (LavaTM Ultimate Restorative), bonded together with adhesive cements using three different luting protocols (total-etch; self-etch; self-adhesive).

**Material and Methods:**

Thirty cylinders were milled from resin nano ceramic blocks with CAD/CAM technology. The cylinders were subsequently cemented to the exposed dentin of 30 bovine permanent mandibular incisors. The specimens were assigned into six groups of five teeth each according to luting procedure and dentin pretreatment. In the first two groups (A1, A2) 10 cylinders were cemented using a total-etch protocol; in groups B1 and B2, 10 cylinders were cemented using a self-etch protocol; in groups C1 and C2, 10 cylinders were cemented using a self-adhesive protocol; in groups A1, B1 and C1 the dentinal surface was also treated with glycine powder. All cemented specimens were submitted to a shear bond strength test. Statistical analysis was performed with Stata 9.0 software.

**Results:**

ANOVA showed the presence of significant differences among the various groups (*P* <0.0001).

**Conclusions:**

Glycine did not change the different bond strength demonstrated by the various luting protocols tested. Conventional resin composite cements used together with a self-etch adhesive reported the highest values. However the use of glycine seems to increase the bond strength of self-adhesive resin cements.

** Key words:**Adhesive cements, CAD/CAM, glycine, luting system, resin nano ceramic, shear bond strength.

## Introduction

Patients’ needs and desires and developments in adhesive dentistry have made the use of all-ceramic restorations increasingly frequent, particularly silica-based ones such as crowns, inlay-onlays and laminate veneers (1). Therefore to improve the impression and casting procedure steps and to produce indirect restorations faster and easier, without the need for provisional restorations and dental laboratories, computer aided design/computer-aided manufacturing (CAD/CAM) systems are commonly used (2). Lava Ultimate Resin Nano Ceramic (RNC) (3M ESPE, St. Paul, MN, USA) is a new composite/nanoceramic material for CAD/CAM manufacturing. This material allows the possibility to use composite materials to characterize and adjust the restoration after milling. Unlike conventional ceramic restorations, customization and glaze firing is neither necessary nor possible with RNC restorations. This opens up the opportunity for intraoral individualization and adaptation of the restorations (3).

Industrially prefabricated CAD/CAM restorations are polymerized by standardized methods, improving material properties, in particular predictability and consistency. Comparing these machinable prostheses to laboratory-handmade restorations, it has been advocated that, due to a highly homogeneous quality crystalline content, the bond strength to hard tooth tissues and the clinical longevity of these CAD/CAM restorations have been increased. In contrast, conventional manual polymerization and processing is greatly influenced by the operator and can cause a high level of variations (4). However, to achieve a long duration of the restoration and therefore its long-term success, durable bond strength between the tooth and the restorative material is fundamental (5).

The literature is unclear on which cement, ceramic, conditioning treatment, and dentine bonding agent produce the highest bond strength. Resin composite cements are used to lute conventional metal crowns, fixed partial dentures, ceramic crowns, and veneers and to repair fractured metal ceramic restorations (6). Resin cements have been selected for their advantageous mechanical and adhesive properties (7). Bond strength to ceramic material is influenced by the composition of the ceramic substrate as well as by mechanical and chemical interaction between substrate and bonding agent (8).

Various studies evaluated the effect of different pretreatments on adhesion between restorative materials and dentin. In order to ensure bond strength, air-polishing devices have been previously reported to increase roughness of both dental hard tissues and restorative materials (9). Amongst these polishing systems, sodium bicarbonate may be disadvantageous as a pretreatment prior to dentin bonding (10). On the other hand, glycine powder is widely used for dentin pretreatment, thus not demonstrating a significant loss of dentin bonding (11).

The purpose of this study was to evaluate the effect of glycine pretreatment on the shear bond strength between dentin and a CAD/CAM resin nano ceramic material (LavaTM Ultimate Restorative), bonded together with adhesive cements using three different luting protocols (total-etch; self-etch; self-adhesive).

## Material and Methods

The specifications of materials tested are listed in  Table 1.

**Table 1:**
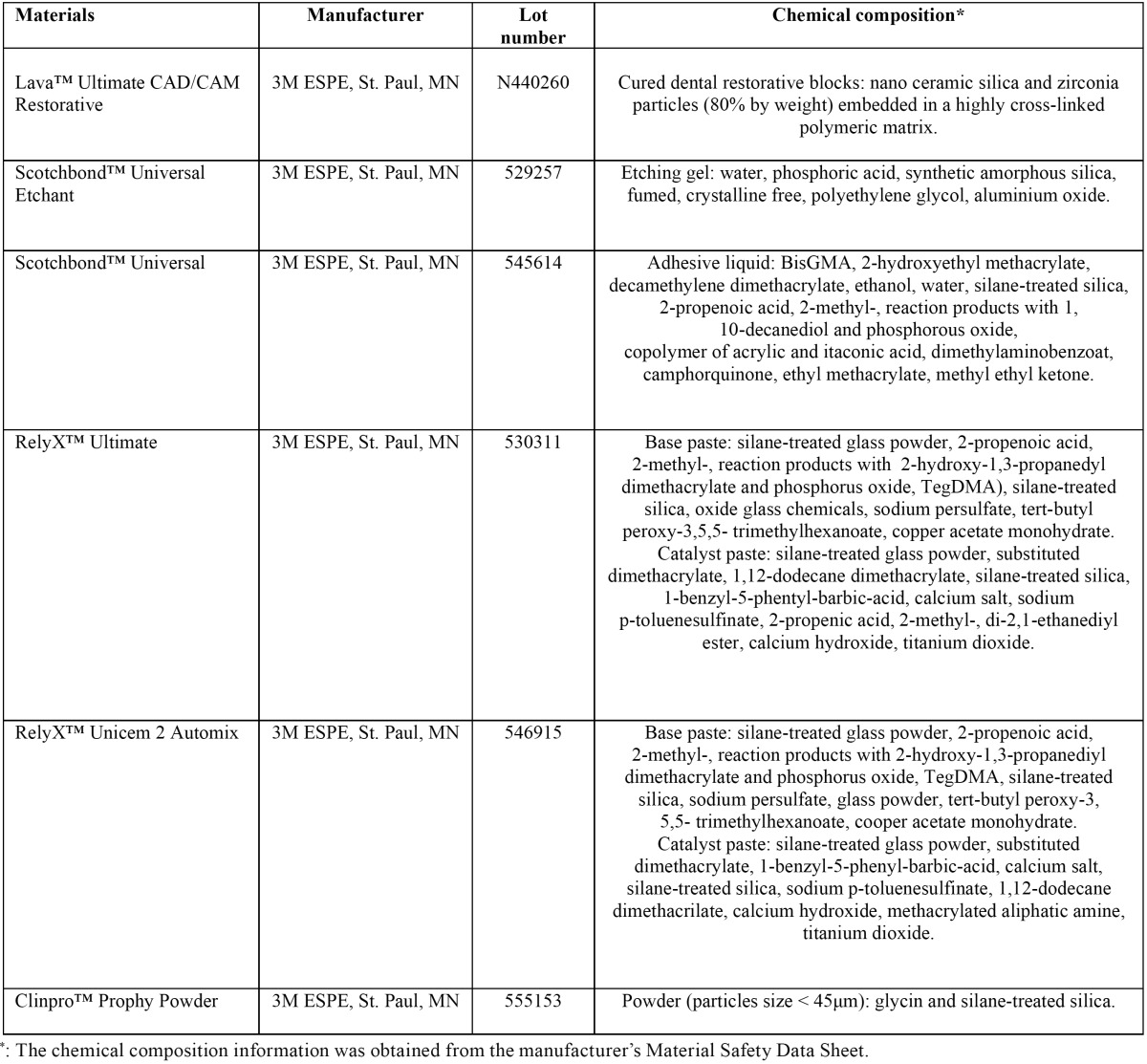
Characteristics of the materials used.

-Specimens’ preparation

In the present *in vitro* study, 30 cylinders (5 mm in diameter, 3 mm thick) were designed with CEREC Software 4.2 platform (Sirona Dental GmbH, Salzburg, Austria) and obtained by milling from resin nano ceramic blocks for CAD/CAM (LavaTM Ultimate Restorative, 3M ESPE, St. Paul, MN, USA) with CEREC MC XL (Sirona Dental, Salzburg, Austria).

The cylinders were subsequently cemented to the exposed dentin of 30 bovine permanent mandibular incisors freshly extracted and stored in a 0.1% (wt/vol) thymol solution, which were used as a substitute for human teeth. Our study was approved by ethics committee of University of Pavia. The criteria for tooth selection included intact buccal enamel with no crack caused by extraction, the absence of caries and adequate dimension of the crown. The teeth were cleansed of soft tissue remnants and debris with periodontal curettes, stored in the tymol solution for one week and later in saline solution at room temperature until testing. Then the roots of the teeth were embedded in self-curing, fast-setting acrylic resin (Rapid Repair, DeguDent GmbH, Hanau, Germany). Specially fabricated cylindrical Teflon mould with an internal diameter of 14 mm were filled with the acrylic resin and allowed to cure, thus encasing each specimen while allowing the buccal surface of dentin to be exposed. Each tooth was oriented so that its labial surface was parallel to the shearing force. The buccal enamel was removed using a high-speed carbide rotary instrument (# H21L.314.014; Komet, Lemgo, Germany) under copious water irrigation, to expose midcoronal dentin. The exposed dentin surfaces were finished off with an automated polishing machine (APL-4; Arotec S.A. Ind Com, Cotia, SP, Brazil) with a 600-grit silicon carbide abrasive paper (SiC) disks for 5 seconds, to obtain a flat and uniform dentin surface and reduce any micromechanical interlocking that could affect the real bonding influence of the tested adhesive cements. Before cementation, the dentin surface was rinsed and treated for 1 minute with a cotton pellet impregnated with Tubulicid Blue (Dental Therapeutics AB, Saltsjo-Boo, Sweden) without fluorine. The surface was then rinsed and dried before cementation; the labial surface of each incisor was cleaned for 10 seconds with a mixture of water and fluoride-free pumice in a rubber-polishing cup with a low-speed handpiece. The dentine surface was rinsed with water to remove pumice or debris and then dried with an oil-free air stream. The bonding surface of each cylinder too was treated with alcohol and rinsed with water to remove oil debris contained in the milling liquid.

-Cementation procedures

**Figure 1:**
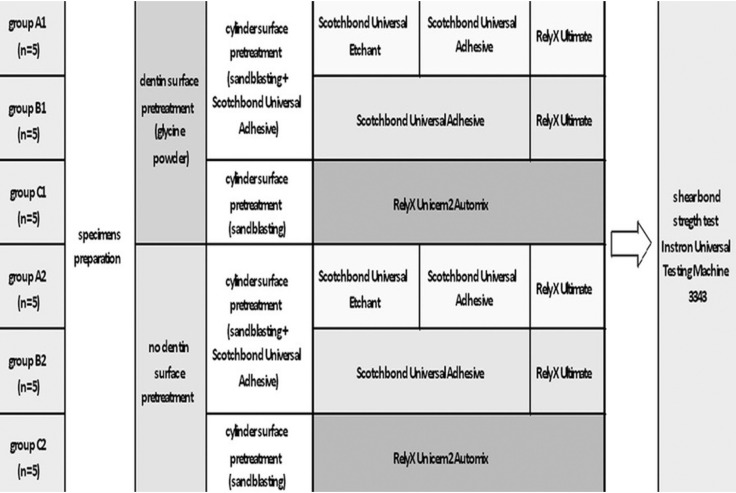
Flow chart of the whole experimentation.

The specimens were randomly assigned into 3 groups of 10 teeth each according to different luting procedures (total-etch, self-etch and self-adhesive). Each group was then divided into two subgroups of five teeth each according to different dentin surface pretreatment (Fig. 1). One operator carried out all procedures to maximize standardization.

GROUP A1 (Gly-ULT-TE): Cylinders were cemented on the dentin surface, which had been acid-etched with Sco35% phosphoric acid (3M ESPE, St. Paul, MN, USA) for 15 seconds (the etched substrates were rinsed with water and gently air dried to remove excess water) and treated with Scotchbond Universal Adhesive (3M ESPE, St. Paul, MN, USA), with adhesive resin cement (RelyXTM Ultimate/3M ESPE, St. Paul, MN, USA). The dentin surfaces of the specimens were also pretreated with glycine powder (ClinproTM Prophy Powder/3M ESPE, St. Paul, MN, USA) for 30 seconds using a Prophyjet device, then rinsed and gently air-dried before etching.

GROUP B1 (Gly-ULT-SE): Cylinders were cemented on the dentin surface, which had been treated with Scotchbond Universal Adhesive, with the adhesive resin cement (RelyXTM Ultimate). The dentin surfaces of the specimens were also pretreated with glycine powder (ClinproTM Prophy Powder) for 30 seconds using a Prophyjet device, then rinsed and gently air-dried before adhesive application.

GROUP C1 (Gly-U2-SA): Cylinders were cemented on the dentin surface with the dual polymerization resin luting agent RelyX TM Unicem 2 Automix. The dentin surfaces of specimens were also pretreated with glycine powder (Cleanpro Prophy Powder - 3M ESPE) for 30 seconds using a Prophyjet device, then rinsed and gently air-dried before luting agent application.

GROUP A2 (ULT-TE), B2 (ULT-SE) and C2 (U2-SA): the same procedures were used as in Group A1, B1 and C1 but the dentin surfaces were not pretreated with glycine powder.

In all groups the bonding surface of each resin nano ceramic cylinder was airborne-particle abraded (BASIC Professional IS, Renfert, Hilzingen, Germany) with 50-μm aluminum oxide particles under a pressure of 2.5 atm. Each sample was then air-cleaned to remove any debris. Scotchbond Universal Adhesive was applied in a thin layer (excessive resin was removed with air) on the bonding surfaces of the cylinder and the dentin with a microbrush according to the manufacturer’s instructions.

In groups C1 and C2 the bonding surface of the cylinders were neither sandblasted nor treated with adhesive (according to the manufacturer’s instructions of Relix Unicem 2).

A vinyl ring with an internal diameter of 4.5 mm was applied under the dentin surface to standardize the adhesion area.

During cementation, a thin layer of cement was applied and distributed to the bonding surface of the cylinders by means of a Hideman spatula. On each specimen, five surfaces were identified: mesial, lingual, distal, buccal, and occlusal. As suggested by the manufacturer, every surface was light-polymerized for 20 seconds at a light intensity of 1000 mW/cm2 using a LED curing light in softstart-polymerization mode (Celalux 2 High-Power LED curing-light, Voco GmbH, Cuxhaven, Germany). The power output (light intensity) of the LED was measured with a Cure Rite radiometer (Caulk-Dentsplymod. 644726, Konstanz, Germany).

All samples were stored in distilled water at room temperature for 24 hours.

-Shear bond strength testing

The specimens were stored in water physiological solution at room temperature for 24 hours after cementation. After storing, the specimens were all submitted to a shear bond strength test to check the strength of adhesion between the two substrates, dentin and resin nano ceramic. This test is defined as a test in which an adhesive agent connects two materials and loaded in shear until separation occurs (12). Specimens were placed in a universal testing machine (Model 3343, Instron Corporation, Norwood, MA, USA). Specimens were secured in the lower jaw of the machine so that the bonded cylinder base was parallel to the shear force direction. Specimens were stressed in an occluso-gingival direction at a crosshead speed of 1 mm/min (13). The maximum load necessary to debond was recorded in Newton (N) and calculated in MPa as a ratio of Newton to surface area of the cylinder. The calculated shear bond strength was determined by dividing the strength at which bond failure occurred by the bonding area (12).

After the testing procedure, the fractured surfaces were examined with an optical microscope (Stereomicroscope SR, Zeiss, Oberkochen, Germany).

To maximize standardization, the same operator prepared the specimens and conducted the tests.

-Statistical analysis 

Statistical analysis was performed with Stata 9.0 software (Stata, College Station, Texas, USA). Descriptive statistics, including the mean, standard deviation, median, and minimum and maximum values were calculated for all groups.

The normality of the data was calculated using the Kolmogorov-Smirnov test. Analysis of variance (ANOVA) was applied to determine whether significant differences in debond strength values existed among the groups. Tukey’s test was used as post-hoc. Significance for all statistical tests was predetermined at *P* < 0.05.

## Results

**Figure 2:**
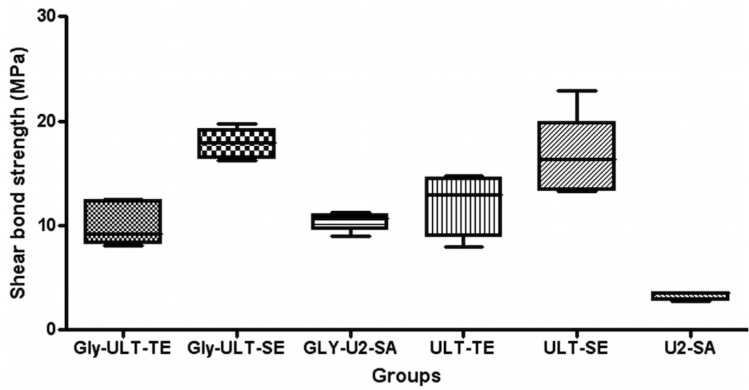
Shear bond strength Box Plot (MPa) of the different groups.

Descriptive statistics are presented in  Table 2. ANOVA showed the presence of significant differences among the various groups (*P*<0.0001). As showed in figure 2, post-hoc Tukey testing showed that the highest shear strength values (*P*<0.001) were reported in groups B1 (Gly-ULT-SE) and B2 (ULT-SE), and no significant differences were detected between the two groups (*P*>0.05).

**Table 2:**
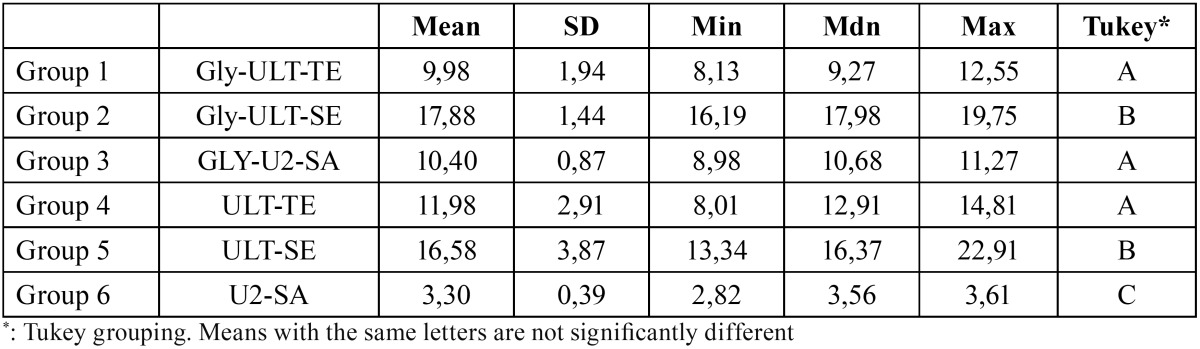
Descriptive statistics (MPa) of the different groups.

Significantly lower (*P*<0.001) shear strength values were showed in groups A1 (Gly-ULT-TE), C1 (Gly-U2-SA) and A2 (ULT-TE). No significant difference was found among the three groups (*P*>0.05). The lowest data (*P*<0.001) were recorded in group C2 (U2-SA).

## Discussion

Increasing demand for esthetic restorations has led to greater use of all ceramic materials because of their improved biocompatibility and optical properties, compared with metal-ceramic restorations (14). Advances in computer-aided design (CAD) and computer aided manufacturing (CAM) systems are providing new options for dentistry, creating an alternative to the conventional impression and casting technique for producing dental restorations (15). Lava Ultimate Resin Nano Ceramic (RNC) blocks are innovative new CAD/CAM materials that make it possible to achieve superior esthetic results in easy steps. The blocks are made of nano ceramic particles embedded in a highly cured resin matrix. Therefore, composite materials can be used to characterize and adjust resin nano ceramic restorations after milling. The milled RNC restorations can be individualized intra-orally or extra-orally, either before or after insertion (3).

In recent years, researchers have tried to achieve a more effective and longer-lasting adhesion between restorative materials and dental substrate. The adhesive techniques are based on research on the hybrid layer and on chemical and mechanical adhesion. Some researchers have attempted to shorten the application time and reduce the number of steps, creating new generations of materials and improving their quality (16). A requirement for the successful function of a CAD/CAM restoration is adequate adhesion between ceramic and tooth substance (17). The aim of this study was to investigate the influence of glycine pretreatment on the shear bond strength between dentin and Lava Ultimate Resin Nano Ceramic (RNC) block, bonded together with three different luting protocols (total-etch; self-etch; self-adhesive).

Although it is preferable to use extracted human teeth for bonding research (18), it has become increasingly difficult to obtain such samples for laboratory studies in Italy. To compare data from the current study with that reported in previous bovine enamel bond strength tests, bovine teeth were used as a substitute for human teeth in the current study. Bovine teeth have some advantages, as they are easy to obtain in large quantities, are in good condition and have less composition variables than human enamel (19). Bovine teeth also have large, flat surfaces and are unlikely to have undergone prior caries challenges that could affect the test result. The mineral distribution within the carious lesions in bovine teeth is reportedly similar to that found in human teeth, and the structural changes that occur in human and bovine teeth are also similar (19).

For dental hard tissues pretreatment glycine powder was selected in this study. Sodium bicarbonate air-polishing powder demonstrated to negatively affect dentin bonding creating a considerably thicker smear layer compared to glycine. Contrariwise the use of glycine powder did not result in a significant loss of dentin bonding performance (11).

For the cementation procedure, three different luting protocols have been evaluated in this study: total etch/etch and rinse protocol (groups A1 and A2: Sco35% phosphoric acid + Scotchbond Universal Adhesive + RelyXTM Ultimate conventional resin cement); self etch protocol (groups B1 and B2: Scotchbond Universal Adhesive + RelyXTM Ultimate conventional resin cement) and self-adhesive protocol (groups C1 and C2: RelyX TM Unicem 2 Automix self-adhesive resin cement). These three different cementation techniques have been selected according to contemporary adhesive systems classification into etch-and-rinse and self-etch adhesives (20,21). While the etch-and-rinse approach requires a separate acid-etch step to promote dentin and enamel demineralization before monomer infiltration, demineralization and infiltration occur simultaneously in the self-etch approach, although with no perfect synchronism (22). In addition, the separate etch-and-rinse step completely removes the smear layer, while the combined etch and bonding step in self-etch adhesive systems only partially dissolve the smear layer. Complete removal of the smear layer may allow for more intimate contact of the hydrophilic primer and hydrophobic bonding agent to the tooth. This allows the characteristic micro retentive resin tags and a hybrid layer to be formed (23).

Many articles related to adhesive procedures used for the cementation of ceramic to tooth structure have shown that the presence of a hybrid layer between adhesive resin and dentin seems to adequately seal the dentinal tubules and allows a cellular reorganization of the pulpal tissues (24,25). In the present study, all the tested cements are based on adhesive procedures, which determine the formation of the hybrid layer and lead to the creation of a stronger link between dental structure and composite cement.

In general two different types of resin composite cements exist: the conventional and the self-adhesive resin composite cements. These two different types of cements have been both evaluated in our study. To bond CAD/CAM restoration to dentin, RelyXTM Ultimate conventional resin cement was used together with a etch and rinse adhesive or with a self etch adhesive; while RelyXTM Unicem 2 Automix self-adhesive resin cement was used alone.

Since they do not necessitate any pre-treatment of tooth substrate, self-adhesive resin composite cements have been developed (26). Self-adhesive resin composite cements contain acid monomers, resulting in an initial lower pH value for the infiltration into the demineralised collagen network (27).

In our study, without glycine application, significant differences were found between conventional and self-adhesive resin cements. The lowest shear bond strength values were recorded in group C2; i.e. Lava Ultimate Resin Nano Ceramic (RNC) disks bonded to dentin with self-adhesive cements and no dentin pretreatment. This is in accordance with a study by Stawarczyk *et al.*, which reported lower tensile bond strength with self-adhesive resin composite cements to polymeric crowns, compared to the bonding with conventional resin cements (28). However the bond strength of these resin composite cements is highly variable. While some products have equal bond strength of self-adhesive resin cement to dentin, other products show an inferior bond to enamel (29). The success of the restoration depends not only on the bond between tooth and resin cement, but also on the bond between restoration and resin composite cement. According to some Authors to achieve a resistant bond, further conditioning of the restoration material is needed (30).

Therefore, comparing conventional resin cements used together with a etch and rinse adhesive or with a self etch adhesive without dentin pretreatment, significantly higher bond strength values were recorded for the self etch protocol. Group B2 (ULT-SE) recorded significantly higher shear strength values than groups A2 (ULT-TE) and C2 (U2-SA). These results are in accordance with a study by Flury *et al.* (31).

As regards the effect of glycine pretreatment on shear bond strength values between new CAD/CAM materials and dentin recent studies reported significant higher values for glycine compared to no pretreatment protocols (32). In our study, after glycine application no significant differences were reported between group A1 (Gly-ULT-TE) and Group C1 (Gly-U2-SA). Both groups showed significantly lower shear strength when compared with a self-etch protocol; i.e. group B1 (Gly-ULT-SE). Glycine application has not significantly influenced shear strength values of ULT-TE (groups A1 and A2) and ULT-SE (groups B1 and B2). On the contrary, glycine application raised bond values of U2-SA (groups C1 and C2).

Within the limitations of this *in vitro* study, pretreatment with glycine did not change the different bond strength demonstrated by the various luting protocols tested. Conventional resin composite cements used together with a self-etch adhesive reported the highest values. However the use of glycine seems to increase the bond strength of self-adhesive resin cements.
